# High risk of malnutrition associated with depressive symptoms in older South Africans living in KwaZulu-Natal, South Africa: a cross-sectional survey

**DOI:** 10.1186/s41043-015-0030-0

**Published:** 2015-10-19

**Authors:** I. Naidoo, Karen E. Charlton, TM Esterhuizen, B. Cassim

**Affiliations:** 1 Department of Geriatrics, University of KwaZulu-Natal, Durban, South Africa; 2 School of Medicine, Faculty of Science, Medicine and Health, University of Wollongong, Wollongong, Australia; 3 Centre for Evidence Based Health Care, Department of Interdisciplinary Health Sciences, Faculty of Medicine and Health Sciences, Stellenbosch University, Stellenbosch, South Africa

**Keywords:** Nutrition screening, Mini Nutritional Assessment, Elderly, Urban, African

## Abstract

**Background:**

Malnutrition contributes to functional and cognitive decline in older adults, which results in decreased quality of life and loss of independence. This study aimed to identify determinants of nutritional risk among community-dwelling adults in KwaZulu-Natal, South Africa.

**Methods:**

A cross-sectional survey was undertaken in 1008 subjects aged 60 years and over who were randomly selected by systematic sampling. Demographics, socioeconomic data and self-reported history of medical conditions were recorded. The Mini Nutritional Assessment-Short Form (MNA-SF) was used to screen for nutritional risk, and the Centre for Epidemiologic Studies Depression scale was administered to all subjects. Descriptive statistics and the Pearson chi-square and Kruskal-Wallis tests were used for statistical analysis. Logistic regression modelling determined predictors of nutritional risk.

**Results:**

Of the 984 participants (mean age = 68.8 ± 7.4 years; range 60–103 years) who completed the MNA-SF, 51 % were classified as having a normal nutritional status, 43.4 % at risk for malnutrition and 5.5 % classified as malnourished. Men were more likely to be either at risk for malnutrition or be malnourished than women (*p* = 0.008), as were subjects with a monthly household income of ≤R1600 per month (~133 USD) (*p* = 0.003). In logistic regression models, depressed people were 2.803 (*p* < 0.001) times more likely to be at risk or be malnourished than those not depressed.

**Conclusion:**

A high prevalence of risk of malnutrition was identified in older South Africans living in an urban area with poor infrastructure. Further investigations are warranted to determine whether the higher prevalence of depressive symptomatology in nutritionally at risk individuals is a determinant or a consequence of malnutrition, in order to develop targeted nutritional interventions in this age group.

## Background

Worldwide, there is a demographic transition with a rapidly increasing proportion of persons aged 60 years and over. South Africa (SA) has the highest percentage of older persons in sub-Saharan Africa, numbering 3.8 million persons or 7.6 % of the total population in this age group with a projected increase to 4.24 million (9.5 % of population) by the year 2015 [1].

In SA, as in many other developing countries undergoing the socio-demographic transition, health challenges that face the older sector of the population include an increased prevalence of non-communicable diseases, accompanied by a functional and cognitive decline, all of which are aggravated by the dynamic socioeconomic environment in the country [2]. The migration of younger members of rural households to cities in search of employment, combined with the effects of international migration and the pandemic of HIV/AIDS in the context of widespread poverty, has resulted in a change in family structures and an erosion of traditional support systems for vulnerable older persons [1]. As a legacy of the 40 years of apartheid in the country, more than half of older Africans (58 %) have had no formal education while 13 % of elderly headed households consisting of a single room, a backyard shack or an informal structure [1]. Notably, the HIV/AIDS pandemic has resulted in a significant loss of younger persons, the consequences of which have led to a loss of principal sources of financial and material support for older persons. This has resulted in the preponderance of multigenerational households that are headed by older women, who are often primary caregivers for their grandchildren [1].

Given the socioeconomic hardships of older Africans, it is not surprising that this group may be at particular nutritional risk. Even in high-income countries, malnutrition is a frequent problem in persons aged 65 years and older [3]. Global estimates of malnutrition indicate a prevalence of 22.8 %, with considerable differences between health and residential settings (rehabilitation, 50.5 %; hospital, 38.7 %; residential aged care, 13.8 %; community, 5.8 %) [3]. In community-dwelling older adults, an additional 32 % of this group is classified as at risk of malnutrition [3]. It is well documented that socioeconomic status, physiological changes of ageing, the burden of chronic diseases and medications [4], together with a decline in functional status, and psychosocial and psychiatric factors adversely influence nutritional status [5]. In turn, malnutrition also contributes to functional [6] and cognitive decline, sarcopenia, diminished immunity, an increased susceptibility to disease as well as a decreased quality of life [7] and an overall higher mortality [8, 9].

Few studies have investigated the nutritional status of older Africans [10, 11]. Between 43 and 50 % of elderly headed South-African households have been reported to experience food poverty whereby they are unable to afford a basic subsistence diet to meet the needs of household members [12]. Rural households and those with a higher number of occupants were more likely to experience food poverty compared to those in urban areas and those comprising only one or two people.

While a number of different methods have been used to assess nutritional status, including anthropometry, biochemical indicators and dietary intake, there is no single ideal measurement for defining malnutrition in the elderly. Anthropometric measures include body mass index (BMI) and mid-upper arm circumference (MUAC). The accuracy of conventional BMI measurement may be compromised due to changes in body composition and kyphosis in older persons [13], while the MUAC is a relatively insensitive measure. Specialized laboratory services required for the biochemical and haematological biomarkers related to malnutrition are often prohibitively expensive and not practical for community-based studies, while dietary intake assessments rely on memory recall from older adults.

The Mini Nutritional Assessment (MNA) comprises four domains that include anthropometry, general assessment, a short dietary assessment and a subjective health assessment. The MNA has been validated in older persons in many countries [14] and has been shown to correlate with clinical indicators and detailed biochemistry. The MNA has a high sensitivity and specificity and is able to detect an increased risk of malnutrition, even when albumin and BMI are in the normal range. The MNA-Short Form (MNA-SF) was subsequently developed to allow a two-step process in low-risk populations, while retaining the validity of the full MNA [15]. The short form version takes 5 min to complete and has been shown to be a reliable and efficient screening tool for community-dwelling elderly patients.

KwaZulu-Natal (KZN), one of the nine provinces in SA, is home to 19.6 % of the total number of persons aged 60 years and over in the country. Most older South Africans subsist on a government old age pension [16] which was R1010 (approximately 84 USD) per month at the time of the current survey. The present study was undertaken to identify the risk of malnutrition in older persons in KZN and to determine its association with other health parameters.

## Methods

### Study design and sampling

A cross-sectional design was used for this study which was conducted in the Inanda, Ntuzuma and KwaMashu (INK) areas of KwaZulu-Natal, SA. According to the 2001 general census [17], 18,812 persons aged 60 years resided in the study area. Using a conservative prevalence estimate of 50 % for a chronic disease, disability or impairment, a sample size of *n* = 1010 was estimated, using a confidence level of 95 % and a precision level of 3 %. A two-staged sampling method was used. Firstly, the sample was distributed proportionately within the wards of Inanda, KwaMashu and Ntuzuma and within formal and informal dwellings in each of the three areas. Thereafter, systematic sampling was used at the level of the street/tract to identify respondents who met the inclusion criteria. The starting points were randomly selected and defined using global positioning system (GPS) coordinates for both formal and informal areas. Starting at the predefined points, skip method was used to identify households. The process continued on street or tract (for informal areas) until a maximum of 25 % of the quota for that segment of the ward was obtained, before the process was repeated on another street/tract. At household level, eligible persons who were 60 years and older were identified, and one person was randomly selected using a Kish grid.

Inclusion criteria included age 60 years and over and the ability to speak and understand English or IsiZulu and to give informed consent. A detailed questionnaire was administered by trained fieldworkers and included demographic and socioeconomic data (educational level (classified as none, primary, secondary and tertiary) and household income), as well as information on housing structure, and household composition. The CAGE questionnaire was used to assess risk of alcohol abuse [18]. To determine the presence of chronic diseases, subjects were asked “Has a doctor or other health professional ever told you that you have any of the following?” from a list of common medical conditions.

To assess oral health, a response of very often, often, sometimes and never was recorded in response to the question “In the last six months how often did you have to avoid eating certain foods because of problems with your mouth, teeth or dentures?” The Centre for Epidemiologic Studies Depression (CESD) scale was completed for all subjects, and scores were categorized as normal (<16) or having depressive symptomatology (≥16) [19].

The MNA-SF was used to categorize subjects as having normal nutrition (score >12), being at risk of malnutrition (10–12) or being malnourished (<10) [20]. Physical activity was assessed by asking whether subjects walked outside, worked in their gardens and/or performed light or heavy household tasks. Food insecurity was assessed by a single question “How often does your household run out of food?” Responses provided as “often” and “about half the time” were classified as indicating food insecurity.

Ethical approval was obtained from the Biomedical and Research Ethics Committee of University of KwaZulu-Natal.

### Data analysis

Data were encoded to ensure anonymity and were double captured and entered into a database supported by Epi-Info 2000. SPSS version 15.0 (SPSS Inc., Chicago, Illinois) was used to analyze the data. A two-tailed *p* value of <0.05 was considered as statistically significant. The demographic characteristics of the study population and the nutritional status are described using frequency tables, percentages and 95 % confidence intervals and displayed graphically using bar charts. Quantitative variables are summarized using summary statistics, and the Pearson chi-square and Kruskal-Wallis tests were used to determine the association between the demographic characteristics, socioeconomic status, prevalence of chronic diseases, alcohol intake, physical maintenance scales and oral health, and nutritional status. A backward stepwise logistic regression method based on likelihood ratios, with entry and removal probabilities set at 0.05 and 0.1, respectively, was used to generate models that predicted either being classified as at risk of malnutrition or being malnourished (binary variable) compared to being well nourished. Variables entered into the models were identified as being significant in bivariate analyses.

## Results

Of the 1010 subjects enrolled in the study, two subjects did not meet the age criterion and were excluded. Complete details on the MNA-SF were available in 984 subjects (20 of the 24 individuals without full MNA-SF details were unable to have height and weight measurements taken because of being bedridden or unable to stand). The majority 978 (99.4 %) were African with 6 (0.6 %) being of mixed ancestry. Most (97.1 %) were in receipt of a government old age pension. The demographic and socioeconomic characteristics of the study subjects are shown in Table 1. Fifty-one percent (*n* = 503) of participants were classified as being well nourished, 43.4 % were considered to be at risk of malnutrition (*n* = 427) and 5.5 % as malnourished (*n* = 54). According to BMI categories, 18 (1.8 %) subjects were classified as undernourished (BMI ≤18.5 kg/m^2^), 196 (19.9 %) as desirable (BMI 18.5–24.9 kg/m^2^), 298 (30.3 %) as overweight (BMI 25–29.9 kg/m^2^) and 472 (48.0 %) as obese (BMI ≥30 kg/m^2^). There was no significant difference in categorization of nutritional risk, according to age categories (*X*^2^ = 5.4, df = 10, *p* = 0.857). Men were more likely to be either at risk for malnutrition or malnourished than women (58 % compared to 46.2 %; *Х*^2^ = 9.76, df = 2, *p* = 0.008), as were subjects with a monthly household income of less than or equal to R1600 (approximately 133 USD) compared to those with an income above R1600 (*X*^2^ = 11.714, df = 2, *p* = 0.003), (Tables 2 and 3). Fifty-eight percent of respondents were classified as being food insecure (23.5 % reported often running out of food; 32.5 % reported about half the time); however, these participants did not have a higher prevalence of being at risk of malnutrition compared to those that were food secure. Fifty-eight percent of respondents were classified as being food insecure (23.5 % reported often running out of food; 32.5 % reported about half the time).

**Table 1:** Characteristics of the study population *n* (%)

	Men *n* = 224	Women *n* = 760	Total *N* = 984
Age (mean ± SD years)	68.0 (7.2)	69.0 (7.4)	68.9 ± 7.4
Range	60–91	60–94	60–94
Marital status			
Widowed	52 (23.2 %)	369 (48.9 %)	434 (44.1 %)
Never married	37 (16.5 %)	217 (28.7 %)	258 (26.2 %)
Married	106 (47.3 %)	102 (13.5 %)	211 (21.4 %)
Separated/divorced	13 (5.8 %)	55 (7.3 %)	81 (8.2 %)
Household Income			
<R1600	136 (61.3 %)	505 (66.7 %)	641 (65.5 %)
≥R1600	86 (38.7 %)	252 (33.3 %)	338 (34.5 %)
No. of persons in household			
Median	5.0	5.0	5.0
Range	1–20	1–16	1–20
Number of households with grandchildren	126 (56.3 %)	588 (77.4 %)	735 (72.9 %)
Housing type			
Formal	122 (54.7 %)	440 (58.0 %)	520 (53.6 %)
Informal	101 (45.3 %)	319 (42.0 %)	451 (46.4 %)
Level of education			
No education	51 (22.8 %)	203 (26.7 %)	254 (25.8 %)
Primary level only	95 (42.4 %)	308 (40.6 %)	403 (41.0 %)
Secondary level	77 (34.4 %)	231 (30.4 %)	308 (31.3 %)
Tertiary level	1 (0.4 %)	17 (2.2 %)	18 (1.8 %)

**Table 2:** Demographic and socioeconomic characteristics according to classification of nutritional risk according to MNA-SF *n* (% MNA-SF category)

	Nutritional categories of MNA-SF			*p* value^a^
	Normal	At risk of malnutrition	Malnourished	
Male	42	51.8	6.2	0.008
Female	53.8	41	5.2	
Income <R1600	313 (62.4 %)	282 (66.7 %)	46 (85.2 %)	0.003
Households with >5 persons	244 (48.8 %)	229 (53.8 %)	29 (53.7 %)	0.301
Households with grandchildren	361 (71.8 %)	315 (73.8 %)	38 (70.4 %)	0.740
Informal housing	281 (56.0 %)	257 (60.3 %)	24 (44.4 %)	0.061
Education				
No schooling	137 (27.3 %)	103 (24.1 %)	14 (25.9 %)	0.430
Primary schooling	197 (39.2 %)	189 (44.3 %)	17 (31.5 %)	
Secondary schooling	160 (31.9 %)	126 (29.5 %)	22 (40.7 %)	
Tertiary education	8 (1.6 %)	9 (2.1 %)	1 (1.9 %)	

^a^Pearson chi-square test for differences between categories of MNA-SF for variable

**Table 3:** Association between MNA-SF nutritional categories and behavioural and clinical indicators, expressed as percentage within each MNA-SF category

		Nutritional categories of MNA-SF, %			*p* value^b^
		Normal	At risk of malnutrition	Malnourished	
BMI (kg/m^2^)					
Undernourished	<18.5	0	2.1	16.7	<0.001
Normal	18.5–24.9	14.7	25.1	27.8	
Overweight	25–29.9	32.4	28.8	22.2	
Obese	≥30	52.9	44.0	33.3	
Poor oral health					
Yes		22.3	27.9	16.7	0.056
Alcohol intake^a^					
CAGE score ≥2		42.6	48.0	0	0.118
Social networks					
Attended social meetings					0.057
Walked to the shops					
Yes		90.1	85.5	72.2	<0.001
Worked in the garden					
Yes		50.6	56.1	92.9	0.013
Performed light household tasks					
Yes		69.6	66.3	59.3	0.228
Performed heavy household tasks					
Yes		38.0	32.9	24.1	0.057
Depressed					
CESD score ≥16		37.6	59.7	79.6	<0.001
Diagnosed clinical conditions (*n* (%))					
Hypertension (*n* = 982)		334 (66.5 %)	269 (63.1 %)	34 (63.0 %)	0.535
Diabetes mellitus (*n* = 983)		96 (19.1 %)	83 (19.4 %)	14 (25.9 %)	0.485
Stroke (*n* = 983)		9 (0.9 %)	20 (2.0 %)	1 (1.9 %)	0.033
Arthritis (*n* = 983)		210 (41.8 %)	155 (36.3 %)	155 (36.3 %)	0.005
Tuberculosis (*n* = 983)		6 (1.2 %)	6 (1.4 %)	4 (7.4 %)	0.002

^a^ *n* = 109 answered this question

^b^Pearson chi-square test for difference in variables according to MNA-SF categories

There was no association between nutritional status (MNA category) and having more than five persons in the household (*X*^2^ = 2.402, df = 2, *p* = 0.301), whether there were grandchildren in the household or not (*X*^2^ = 0.602, df = 2, *p* = 0.740), nor with level of education (*X*^2^ = 5.936, df = 6, *p* = 0.430).

Of the 109 subjects (10.8 % of total sample) who reported consuming alcohol, 62 (56.9 %) had a CAGE score of <2 and 47 (43.1 %) a CAGE score of ≥2, but this did not differ across nutritional risk categories (*X*^2^ = 4.28, df = 2, *p* = 0.118). Sixty-three percent of subjects reported attending regular social activities and meetings with a trend for those not participating in social activities being more likely to be malnourished (*X*^2^ = 17.880, df = 10, *p* = 0.057). Subjects who did not walk outside were more likely to be at risk for malnutrition or malnourished compared to those who did (*X*^2^ = 15.5, df = 2, *p* < 0.001). Five hundred and nine subjects (51.7 %) had poor oral health as defined by having to avoid eating certain foods (often or very often) in the past 6 months because of problems with their mouth, teeth or dentures. These participants were not more likely to be at risk for malnutrition/malnourished, compared to those subjects who did not report oral problems (*X*^2^ = 46.2, df = 6, *p* = 0.056).

Based on the CESD, 487 (49.5 %) of subjects had depressive symptoms. Of those characterized as being well nourished, 189 (37.6 %) were depressed; of those at risk of malnutrition, 255 (59.7 %) were depressed; and of the malnourished, 43 (79.6 %) were depressed (*X*^2^ = 66.0, df = 2, *p* = <0.001).

Two logistic regression models were identified. For model 1, variables entered into a backward stepwise model were age, gender, income, household size, poor oral health, walking outside to the shops and performing heavy household tasks. The following variables remained in the final model: gender, income, household size and walking outside to the shops. Males were 1.68 times more at risk than females, those with income below R1600 were 1.43 times more at risk, those with larger household sizes were 1.32 times more at risk and those who do not walk outside to the shops were 1.9 times more at risk than those who did. In model 2, depression was added and the adjusted odds ratio for at risk/malnutrition in depressed people was 2.8 (*p* < 0.001). In this model, household size and income were no longer associated with malnutrition (Table 4).

**Table 4:** Logistic regression models for classification of nutritional risk. Outcome variable is “at risk of malnutrition” and “malnourished”, according to MNA-SF classification

Variables		B	S.E.	Wald	df	Sig.	OR	95 % CI for OR	
								Lower	Upper
Model 1									
Step 4^a^	Gender: male vs female	.521	.157	11.018	1	.001	1.684	1.238	2.291
	Income: <R1600 vs ≥ R1600	.355	.141	6.300	1	.012	1.425	1.081	1.880
	Household (HH) size: ≥5 vs <5	.280	.134	4.396	1	.036	1.324	1.018	1.720
	Not walking outside to the shops (vs yes)	.643	.200	10.308	1	.001	1.901	1.284	2.815
	Constant	−.621	.152	16.787	1	.000	0.537		
Model 2									
Step 5^b^	Gender: male vs female	.592	.163	13.275	1	.000	1.808	1.315	2.486
	Not walking outside to the shops (vs yes)	.460	.210	4.808	1	.028	1.584	1.050	2.389
	Not performing heavy household tasks (vs yes)	.267	.145	3.417	1	.065	1.307	.984	1.735
	Depressed (vs not depressed)	1.031	.135	58.508	1	.000	2.803	2.153	3.650
	Constant	−.656	.117	31.384	1	.000	.519		

^a^Variables entered in step 1: age, gender, income, HH size, poor oral health, walking outside to the shops, performing heavy household tasks

^b^Variables entered in step 1: age, gender, income, HH size, poor oral health, walking outside to the shops, performing heavy household tasks and depression

## Discussion

To our knowledge, this is the largest study to report on the nutritional risk of community-living persons aged 60 years and over from an African country. Using the validated MNA-SF, 43.4 % of participants were considered to be at risk of malnutrition, while 5.5 % had an MNA-SF score that indicates malnutrition. The prevalence of malnutrition is considerably higher than that reported in community-dwelling older populations from higher-income countries [21–24]. In a sample of 22,007 subjects in Spain, a similar prevalence of malnutrition was identified (4.3 %), but a smaller proportion of individuals was characterized as being at risk (25.4 %) [25]. A cross-sectional study conducted in another developing country, India, in 227 participants aged 60 years and over, found that 14 % were malnourished and 49 % were at risk using the full MNA instrument [26] while in Bangladesh, of the 625 subjects interviewed, 26 % of subjects were malnourished and 55 % were at risk [27]. In both these studies, participants had no income, incurred a high burden of disease, resided in multigenerational households and had poor or no education, all of which were reported as likely contributing factors to low MNA scores. Similarly in a Ugandan study that used BMI to assess nutritional status, 33 % of the subjects aged between 60 and 90 years were classified as malnourished [6]. Ugandan older persons also had no income or government pensions, resided in multigenerational homes and had a high burden of disease. In contrast, most of our study subjects were in receipt of a non-contributory government-funded old age pension, which may explain, in part, the lower prevalence of nutritional risk.

Determinants of poor nutritional status are varied and numerous. Several studies have shown that older adults are more vulnerable to malnutrition than younger adults due to the presence of multiple pathologies and associated decreased oral intakes [25–27] while other studies have shown no significant association between age and nutritional status [28, 29]. The lack of an age association with nutritional risk in our sample may be related to the age distribution of our sample being mostly between 60 and 64 years and relatively few older participants.

The overall socioeconomic status of subjects was poor; most participants received a monthly pension of R1010 as their principal or only source of income. Subjects living in households with a cumulative income of <R1600 were more likely to have an MNA-SF score that indicates nutritional risk. This is consistent with findings of other studies conducted in SA [12, 30]. Other socio-demographic characteristics, including household size, living in a multigenerational household and level of education, were not associated with an MNA-SF score.

Decreased physical activity can impact on nutritional status by limiting the ability of an older person to produce, acquire and/or prepare food. In this study, subjects who reported that they did not walk to the shop were more at risk of malnutrition or malnourished, as has been reported by others [6].

The lack of an association between poor oral health and malnutrition is inconsistent with findings from other studies [28, 29, 31]. A Brazilian study found that poor oral health, defined as edentulism without denture-wearing, was more likely to be associated with unfavourable body mass, both underweight (OR = 3.94; 1.14–13.64) and overweight/obesity (OR = 2.88; 1.12–7.40), taking into account confounders of physical activity and depressive symptomatology [32]. In our sample, poor oral health was self-reported by over half of participants but was not predictive of nutritional risk, presumably because of more important overriding socioeconomic determinants. The South African Demographic and Health Survey [27] reported that 36 % of participants had oral health problems, and these were more common in older persons and those with lower educational and income levels. In a later study, while 76.3 % of South African adults aged 16+ years rated their oral health as good, only 38.1 % of those aged 65+ years rated their oral health as good [28]. In that study, participants who had no education or were unemployed were also more likely to have oral problems. The high prevalence of oral/dental problems in SA is thought to be due to poor access to dental care especially in the public sector, where it is estimated that there are approximately only 0.004 dentists per 1000 population [29].

Depression has been shown to be a major determinant of malnutrition in older adults [33]. Depressive symptomatology was strongly associated with suboptimal nutritional status in the present study, and inclusion of this variable in regression models negated the effect of socio-demographic influences. Whether this is a cause or effect is unclear.

Food insecurity was reported by over half of the study population, as has been reported in another South-African study [34]. Although these households were more likely to have a higher total monthly income, they comprised more household members and had a greater proportion of inhabitants being classified as depressed or being at risk for excessive alcohol intake.

Our study has a number of limitations. Data related to components of the MNA-SF was self-reported and may either over- or under-represent the presence of risk factors included within the screening instrument. Height and weight were measured but not available for 20 participants who were bedridden and who may have been more likely to be nutritionally compromised. Thus, the prevalence of malnutrition may have been under-reported. Lastly, food insecurity was assessed using a single question, and this may have been insensitive in a population of low socioeconomic status.

## Conclusions

In this community-based study, more than half of older adults were considered to be at risk of malnutrition or malnourished. These findings highlight a need for nutritional screening to be included in primary care settings and followed up with appropriate clinical pathways and referral systems, particularly to mental health services.

## References

[1] Lombard A, Kruger E (2009). Older persons: the case of South Africa. Ageing Int.

[2] Ataguba JE, Akazili J, McIntyre D (2011). Socioeconomic-related health inequality in South Africa: evidence from General Household Surveys. Int J Equity Health.

[3] Kaiser M, Bauer JM, Rämsch C, Uter W, Guigoz Y, Cederholm T (2010). Frequency of malnutrition in the elderly: a multinational perspective using the Mini Nutritional Assessment (MNA®). J Am Ger Soc.

[4] Roe D (1994). Medications and nutrition in the elderly. Prim Care.

[5] BAPEN. The executive summary of the ‘MUST’ report [Online]. Available: http://www.bapen.org.uk/must_report.html 2003. Accessed 03/03/2015.

[6] Kikafunda J, Lukwago FB (2005). Nutritional status and functional ability of the elderly age 60–90 years in the Mpigi district of Central Uganda. Nutrition.

[7] Millen B. Preventive nutrition services for the aging population. Springer, 1999: p. 121–132.

[8] Charlton KE, Nichols C, Batterham M, Bowden S, Lambert K, Baronne L (2012). Poor nutritional status of older rehabilitation patients predicts clinical outcomes and mortality at 18 months of follow up. Eur J Clin Nutr.

[9] Charlton K, Batterham M, Bowden S, Ghosh A, Caldwell K, Potter J (2013). A high prevalence of malnutrition in acute geriatric patients predicts adverse clinical outcomes and mortality at 12 months. e-SPEN J.

[10] Charlton K, Kolbe-Alexander TL, JH N (2007). The MNA, but not the DETERMINE, screening tool is a valid indicator of nutritional status in elderly Africans. Nutrition.

[11] Kimokoti RW, Hamer DH (2008). Nutrition, health, and aging in sub-Saharan Africa. Nutr Rev.

[12] Charlton K, Rose D. Nutrition among older adults in Africa: the situation at the beginning of the millennium. Nutrition and Dietetics Unit, Department of Medicine, University of Capetown: Cape Town, South Africa.

[13] Pieterse S, Manandhar M, Ismail S (1998). The nutritional status of older Rwandan refugees. Pub Health Nutr.

[14] Guigoz Y, Vellas B, Garry PJ (1996). Assessing the nutritional status of the elderly: the Mini Nutritional Assessment as part of the geriatric evaluation. Nutr Rev.

[15] Kaiser M, Bauer JM, Rämsch C, Uter W, Guigoz Y, Cederholm T (2009). Validation of the Mini Nutritional Assessment® Short-Form (MNA-SF): a practical tool for identification of nutritional status. J Nutr Health & Aging.

[16] Barrientos, A., Ferreira, M., Gorman, M., Heslop, A., Legido-Quigley, H., Lloyd-Sherlock, P. et al. Noncontributory pensions and poverty prevention: a comparative study of South Africa and Brazil. HelpAge International and Institute for Development Policy and Management, London, UK, 2003.

[17] Magnusson R, Reeve B (2014). ‘Steering’ private regulation? A new strategy for reducing population salt intake in Australia. Sydney Law Review.

[18] Ewing A (1984). Detecting alcoholism: the CAGE questionnaire. JAMA.

[19] Radloff, L., Centre for Epidemiologic Studies, N.I.o.M. Health, Editor, West Publishing Company.

[20] A guide to completing the MNA-SF, Nestle Nutrition Institute. Available at: http://www.mna-elderly.com/forms/mna_guide_english_sf.pdf. Accessed 7/10/2015.

[21] Winter J, Flanagan D, McNaughton SA, Nowson C (2013). Nutrition screening of older people in a community general practice, using the MNA-SF. J Nutr Health Aging.

[22] Nykänen I, Lönnroos E, Kautiainen H, Sulkava R, Hartikainen S (2013). Nutritional screening in a population-based cohort of community-dwelling older people. Eur J Public Health.

[23] Timpini A, Facchi E, Cossi S, Ghisla MK, Romanelli G, Marengoni A (2011). Self-reported socio-economic status, social, physical and leisure activities and risk for malnutrition in late life: a cross-sectional population-based study. J Nutr Health Aging.

[24] Ülger Z, Halil M, Kalan I, Yavuz BB, Cankurtaran M, Güngör E (2010). Comprehensive assessment of malnutrition risk and related factors in a large group of community-dwelling older adults. Clin Nutr.

[25] Cuervo M, Garcia A, Ansorena D, Sanchez-Villegas A, Martinez-Gonzalez M, Astiasaran I (2009). Nutritional assessment interpretation on 22,007 Spanish community-dwelling elders through the Mini Nutritional Assessment test. Pub Health Nutr.

[26] Vedantam A, Subramanian V, Rao NV, John KR (2010). Malnutrition in free-living elderly in rural south India: prevalence and risk factors. Pub Health Nutr.

[27] Ferdous T, Kabir ZN, Wahlin A, Streatfield K, Cederholm T (2009). The multidimensional background of malnutrition among rural older individuals in Bangladesh—a challenge for the Millennium Development Goal. Pub Health Nutr.

[28] Soini H, Routasalo P, Lagstrom H (2004). Characteristics of MNA in elderly home care patients. Eur J Clin Nutr.

[29] Iizaka S, Tandaka E, Sandada H (2004). Comprehensive assessment of nutritional factors in the healthy, community dwelling elderly. Geriatric and Gerontology International.

[30] Oldwage-Theron W, Salami L, Zotor FB (2008). Health status of an elderly population in Sharpeville. South Africa Health SA Gesondheid.

[31] Lamy M, Mojon P, Kalykakis G, Legrand R, Butz-Jorgensen E (1999). Oral status and nutrition in the institutionalized elderly. J Dent.

[32] Tôrres L, Da Silva DD, Neri AL, Hilgert JB, Hugo FN, Sousa ML (2013). Association between underweight and overweight/obesity with oral health among independently living Brazilian elderly. Nutrition.

[33] Feldblum I, Germa L, Castel H, Harm-Boehm I, Bilenko N (2007). Characteristics of undernourished older medical patients and identification of predictors for an undernourished state. Nutr J.

[34] Rose D, Charlton KE (2002). Prevalence of household food poverty in South Africa: results from a large, nationally representative survey. Pub Health Nutr.

